# Enhanced Electrochemical Performance of Binder-Free Fluorine–Vanadium-Doped CoMoO_4_ Nanosheets via In Situ MXene Integration for Energy Storage Applications

**DOI:** 10.1021/acsaem.5c01660

**Published:** 2025-07-22

**Authors:** Monaam Benali, Rasmita Barik, Rui Gusmão, Jan Luxa, Piotr W. Zabierowski, Amutha Subramani, Bing Wu, Zdeněk Sofer

**Affiliations:** Department of Inorganic Chemistry, University of Chemistry and Technology Prague, Technicka 5, 166 28 Prague 6, Czech Republic

**Keywords:** transition metal, doping, energy storage, supercapacitor, hydrothermal, MXene

## Abstract

Designing an affordable device that seamlessly combines efficient electrochemical energy storage with straightforward, robust protocols represents a promising pathway for ushering in the next generation of green power solutions and fostering a sustainable society. In this work, CoMoO_4_, vanadium-doped CoMoO_4_ (V-CoMoO_4_), and fluorine–vanadium-doped CoMoO_4_ (F-V-CoMoO_4_) were synthesized in situ on nickel foam (NF) using a hydrothermal method, followed by thermal treatment, resulting in a hierarchical structure with interconnected nanosheets and open porous channels. V_2_C MXene was used as the vanadium source, which was fully oxidized during the synthesis. This unique architecture is particularly advantageous for supercapacitor applications, as it facilitates efficient electrolyte flow, promotes the formation of oxygen defects that enhance ion transport, and ultimately maximizes electrochemical performances. At a current density of 2.5 mA/cm^2^, the F-V-CoMoO_4_ electrode achieves an areal capacitance of approximately 2250 mF/cm^2^ (900 F/g at 1 A/g), outperforming pristine CoMoO_4_ (180 mF/cm^2^, 72 F/g) and V-doped CoMoO_4_ (810 mF/cm^2^, 324 F/g). An asymmetric supercapacitor is fabricated using an F-V-CoMoO_4_@NF//AC@NF device and PVA/KOH gel electrolyte, showing excellent redox behavior and cycling stability, with 100% capacity retention after 2000 cycles at a current density of 1 Ag^–1^. Moreover, the developed device exhibits a specific energy density of 11.5 Whkg^–1^ and a power density of 225 Wkg^–1^ at a current density of 0.3 A/g. These findings highlight the potential of F–V doping in enhancing the electrochemical properties of CoMoO_4_-based electrodes.

## Graphical abstract

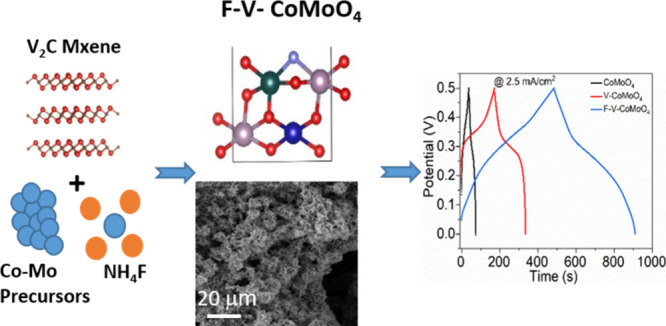

## 1 Introduction

High energy density and long lifespan supercapacitors (SCs) are crucial performance metrics in modern industry, playing a vital role in advancing the green economy,^1^ particularly in response to the growing demands for powering electronics,^2^ electric vehicles, and energy storage solutions.^3–5^ Despite advances, current SC technology, which relies on carbon-based materials or conducting polymers, is mainly limited by low energy density, restricting their widespread adoption. Moreover, recent advances in laser microannealing of Ni-rich layered oxides on flexible polymer substrates have opened pathways for integrating high-performance microcathodes into compact energy storage systems and flexible electronics.^6^ In this context, pseudocapacitive materials, such as hybrid or modified transition metal oxides, which exhibit rapid and highly reversible oxidation/reduction reaction kinetics at or near the electrode surface, have the potential to simultaneously achieve higher energy and power densities.^6–8^ Recently, CoMoO_4_ has drawn significant interest as a SC electrode due to its exceptional catalytic, electrical, and structural properties.^9–11^ Its unique crystalline structure offers remarkable hybrid capacitive properties, attributed to its high surface area, rich intercalation, and excellent ionic conductivity. However, CoMoO_4_’s inherently low electrical conductivity and limited cycling stability may present challenges, making it less suitable as a standalone material for highly stable and long-lasting electrodes.^12^ A promising strategy to address the aforementioned challenges involves doping CoMoO_4_ or fabricating CoMoO_4_-based composites.^13^ These approaches enable the exploitation of the intrinsic properties of the host material and tuning of both its morphology and structure. Such modifications can introduce additional electrochemically active sites and enhance ion diffusion kinetics by shortening the ionic transport pathway, factors that are beneficial for such an electrochemical energy storage system. Notably, the Ni-doped CoMoO_4_ electrode showed a higher specific capacitance value, superior rate capability, and lower pristine CoMoO_4_ due to synergistic multimetal redox transitions and enhanced conductivity. Similarly, the incorporation of lanthanum (La) elements into the CoMoO_4_ lattice with a nanocube structure demonstrated a synergistic effect between the metal oxide and La dopant, leading to improved electrochemical performance. It achieved a specific capacitance of 1552.7 F/g at a scan rate of 5 mV/s, approximately four times higher than the pristine electrode, and maintained about 98% retention with 99% Coulombic efficiency.^14^ Moreover, heterostructures, such as CoMoO_4_@CoP grown in situ on boron-doped graphene aerogel,^15^ CoMoO_4_@NiS_2_ core–shell,^16^ NiCo@NiOOH@CoMoO_4_ core–shell,^7^ and so on, have demonstrated significant enhancement in electrochemical energy storage performance. These studies emphasize the crucial role of the engineered morphology and structure in optimizing the electrochemical characteristics of CoMoO_4_ electrodes. Particularly, features including high dislocation densities, lattice distortion, abundant oxygen vacancies, and a rich redox behavior contribute to enhanced performance.^14,15^ In addition, the reduction in charge transfer resistance (*R*_ct_) leads to an increased number of electrochemically accessible active sites and a larger surface area,^17^ thereby improving ion transport and electrical conductivity. These improvements ultimately result in superior capacitance and high stability of the modified CoMoO_4_ electrode.^18–20^

In recent studies, MXene-CoMoO_4_ composites have exhibited synergistic enhancements in charge storage, suggesting the benefits of combining layered 2D materials with transition metal oxides.^20^

In this regard, we conducted a comprehensive investigation of the properties of F-V-CoMoO_4_ composites, which were grown in situ on Ni foam using a two-step hydrothermal method, followed by an annealing process. These composites are subsequently employed as high-performance supercapacitor electrodes. The F-doping and unique composite are designed to enhance electrochemical performance, resulting in promising properties for advanced energy storage applications. A novel synthesis approach was adopted to develop high-performance CoMoO_4_-based electrodes by combining V_2_C MXene, cobalt molybdate precursors, and NH_4_F. Remarkably, during synthesis, the V_2_C MXene is completely oxidized, without traces of the original MXene or any VO_*x*_ phase detected. Instead, the process induced the formation of unique F-V-CoMoO_4_ nanosheets, potentially featuring finely dispersed VO_*x*_ species and/or incorporated V atoms, which significantly enhanced the electrochemical properties of CoMoO_4_. Vanadium­(V) has a small ionic radius and can adopt multiple oxidation states, each associated with a different ionic radius, which promotes the electronic transitions that are crucial for the electrochemical charge storage reaction. Moreover, its synergistic interaction with other transition metals can tune the overall electronic structure, favoring electron transfer and inducing abundant electroactive sites.^21,22^ Additionally, the aim of incorporating fluorine (F) into the CoMoO_4_ matrix is to tailor its surface structure and induce the formation of oxygen defects, the modification that has been proven effective in enhancing electrochemical performance.^23,24^

Supercapacitors require high-performance electrode materials with excellent electrical conductivity and ion transport properties. In this work, we investigate the impact of vanadium­(V) and fluorine (F) doping on the electronic structure and ion transport properties of CoMoO_4_ by using DFT simulations with Quantum ATK. Our results indicate that doping with V and F significantly enhances the density of states (DOS) near the Fermi level and lowers the effective potential barrier. The calculated work function decreases from 6.3 eV for pristine CoMoO_4_ to 5.6 eV for doped CoMoO_4_, indicating improved electron emission properties. These improvements make V- and F-doped CoMoO_4_ a promising candidate for next-generation energy storage devices.

## 2 Experimental Details

### 2.1 Materials

All chemicals used in this work were of analytical reagent grade and were used without further purification. Cobalt chloride hexahydrate (100%) and potassium hydroxide (KOH) were provided by Lach-Ner, while sodium molybdate dihydrate (99.5%) and ammonium fluoride were purchased from Sigma-Aldrich. Urea (99%) was obtained from the PENTA Company. The nickel foam substrate (99.9%) had a thickness of 0.3 mm.

### 2.2 Synthesis of Pristine CoMoO_4_, V-Doped CoMoO_4_, and F-V-Doped CoMoO_4_

#### 2.2.1 Pristine CoMoO_4_

1 g of cobalt chloride hexahydrate (CoCl_2_·6H_2_O), 0.96 g of sodium molybdate dihydrate (Na_2_MoO_4_·2H_2_O), and 0.152 g of urea were each dissolved separately in 20 mL of deionized water. The solutions were then combined and magnetically stirred for 30 min, forming a uniform purple solution. The above solution was transferred into a 120 mL Teflon-lined stainless-steel autoclave, with a slice of pretreated nickel foam (NF) placed inside. The hydrothermal treatment was carried out at 180 °C for 12 h. The precursor on NF was thoroughly rinsed with deionized water and absolute ethanol to suppress weakly adhered CoMoO_4_ and other impurities on the surface, followed by drying at 60 °C overnight. Finally, the precursor was subjected to calcination in an air atmosphere at 350 °C for 2 h to form crystallized CoMoO_4_.

#### 2.2.2 V-CoMoO_4_

We adopted a similar protocol for the preparation of the V-doped CoMoO_4_ composite. First, 1 g of CoCl_2_·6H_2_O, 0.152 g of urea, and 0.96 g of Na_2_MoO_4_·2H_2_O were separately dissolved in 20 mL of deionized water. Simultaneously, 100 mg of V_2_C MXene was sonicated in 40 mL of deionized water for 30 min. V_2_C MXene was synthesized, as reported in the previous work.^25^ The solutions were then combined and magnetically stirred for 1 h. The resulting mixture was transferred to a 120 mL autoclave with a slice of pretreated NF placed inside. The hydrothermal treatment was conducted at 180 °C for 12 h. Thereafter, the NF substrate was thoroughly washed with deionized water and ethanol, dried at 60 °C overnight, and finally calcined in an air atmosphere at 350 °C for 2 h.

#### 2.2.3 Fluorine–Vanadium-Doped CoMoO_4_ (F-V-CoMoO_4_)

The F-doped sample was prepared by following the same procedure as described above. Specifically, 1 g of NH_4_F was dissolved separately in 20 mL of deionized water and then mixed with the previously prepared solutions. The mixture was subjected to the same hydrothermal treatment as outlined in the above protocol.

### 2.3 Characterization

Powder X-ray diffraction (XRD) patterns of the synthesized materials were collected by using a Bruker D8 Advance diffractometer equipped with a Cu Kα radiation source (λ = 1.5406 Å). The instrument was operated at 40 kV and 40 mA, with measurements recorded over a 2θ range of 4–90° and a scanning speed of 2° per minute. The morphology of the materials was examined by scanning electron microscopy (SEM) with an FEG electron source (Tescan Maia dual-beam microscope) at a 5 kV acceleration voltage. The elemental composition of synthesized materials was investigated by energy-dispersive X-ray spectroscopy (EDX) using an X-Max^N^ detector from Oxford Instruments, with a 20 kV acceleration voltage. Samples were directly placed on a C or Cu tape. Raman spectroscopy was performed using a Renishaw InVia spectrometer to identify the characteristic vibrational modes of the samples. Measurements were carried out at room temperature over a spectral range of 100–2000 cm^–1^, utilizing a He–Cd laser with an excitation wavelength of 532 nm. A 20× objective lens was employed to ensure precise focus on the sample, with the laser power set to 5 mW. The specific surface area of the proposed electrodes was determined by using the Brunauer–Emmett–Teller (BET) method. N_2_ adsorption–desorption measurements were carried out with a Quantachrome NOVA Touch 4LX instrument. High-resolution X-ray photoelectron spectroscopy (XPS) was conducted by using a monochromatic aluminum source (1486.7 eV). Comprehensive survey scans were first performed to detect all elements, followed by detailed high-resolution scans of the C 1*s*, Co 2*p*, Mo 3*d*, O 1*s*, V 3*d*, F 1*s*. The in situ grown samples on NF were positioned on a conductive substrate for measurements.

### 2.4 Electrochemical Measurements and Electrode Preparation

An Autolab PGSTAT 204 (Nova, Utrecht, The Netherlands) was used for all electrochemical measurements, including cyclic voltammetry (CV), galvanostatic charge–discharge (GCD), chronoamperometry, and electrochemical impedance spectroscopy (EIS). EIS analysis was performed in the frequency range of 10 mHz to 100 kHz at zero voltage bias. In the three-electrode system, the instate cobalt molybdate on NF was used as a free binder working electrode, platinum (Pt) was used as the counter electrode, and Hg/HgO was used as the reference electrode. All of the electrochemical characteristics were obtained in 6 M KOH aqueous solutions.

Areal capacitance (*C*_areal_), specific capacitance, energy density, and power density are determined from the galvanostatic charge–discharge plots using the following equations:

$$C_{\mathrm{areal}}\;(F\;{\mathrm{cm}}^{-2})=\frac{i\times \Delta t}{A\times \Delta V}$$ 1

$$C_{\mathrm{sp}}=\frac{i\times \Delta t}{m\times \Delta V}$$ 2

$$\mathrm{Energy}\;\mathrm{density}\;({\mathrm{Whkg}}^{-1}),\;E=\frac{1}{2}\frac{C\times V^{2}}{3.6}$$ 3

$$\mathrm{Power}\;\mathrm{density}\;({\mathrm{Wkg}}^{-1}),\;P=\frac{E\times 3600}{\Delta t}$$ 4

where *i* denotes the current, *t* is the discharge time, *A* is the area of electrodes, and Δ*V* is the potential window.

### 2.5 Density Functional Theory Calculations

#### 2.5.1 Computational Methodology

Density functional theory (DFT) calculations were performed using the QuantumATK software package with the generalized gradient approximation (GGA) and the Perdew–Burke–Ernzerhof (PBE) exchange-correlation functional.^26^ To account for long-range van der Waals interactions, Grimme’s DFT-D3 dispersion corrections were incorporated. A plane-wave cutoff energy of 500 eV was used, ensuring sufficient accuracy and convergence of the total energy. The Brillouin zone was sampled using a 3 × 3 × 1 k-point mesh within the Monkhorst–Pack scheme. Convergence tests were conducted to confirm the adequacy of these computational parameters. Doping was introduced by substituting select Co and O atoms with V and F atoms, respectively. Structural relaxation was performed until the total energy converged within 10^–5^ eV. The electronic structure, including the projected density of states, was analyzed to assess the impact of doping on the material’s electronic properties. Work function calculations were carried out using slab models to evaluate changes in surface electronic characteristics. These computational settings and analysis methods ensure a reliable investigation of the doped system’s structural and electronic properties.

## 3 Results and Discussion

### 3.1 Analysis and Electrochemical Performance of the Positive Electrode Materials

Scheme 1 illustrates the schematic representation of F-V-CoMoO_4_ with dual defects synthesized on nickel foam. The doping elements and defect formation were achieved through a hydrothermal method. During the sequential nucleation and crystal growth stages, exfoliated V_2_C MXene sheets undergo hydrolysis, leading to their in situ deposition along with F-CoMoO_4_ precursors on the growth substrate. In this process, oxygen atoms are partially replaced by fluorine, generating oxygen defects sites. Subsequently, the calcined samples promote the crystallization of the CoMoO4 backbone, incorporating MXene-derived vanadium atoms and fluorine modification, thereby enhancing the structural integrity and electrochemical properties of CoMoO_4_. The morphologies of the different synthesized composite materials are characterized using scanning electron microscopy (SEM), as illustrated in Figure 1. Following the hydrothermal growth process, the surface of the Ni foam is uniformly coated with well-aligned CoMoO_4_ nanosheets, as depicted in Figure 1a,b. This uniform coverage indicates a controlled and effective deposition, resulting in a coherent nanosheet across the foam substrate. In Figure 1b, the CoMoO_4_ nanosheets exhibit a highly interconnected arrangement, forming a hierarchical structure with open and porous spaces. This architecture is particularly advantageous for supercapacitor applications, as the open structure facilitates efficient electrolyte flow up, while the large surface area could favor ion transport and maximize electrochemical activity. Similarly, the V-CoMoO_4_ sample showed an interconnected sheet structure, making it difficult to distinguish any morphological differences from the pristine sample. Based on Figure 1e,f, the nanosheets exhibit interconnectivity, forming a well-organized structure with a porous texture. This arrangement results in a highly ordered array, contributing to the F-V-CoMoO_4_ composite structural integrity.

**Scheme 1:**
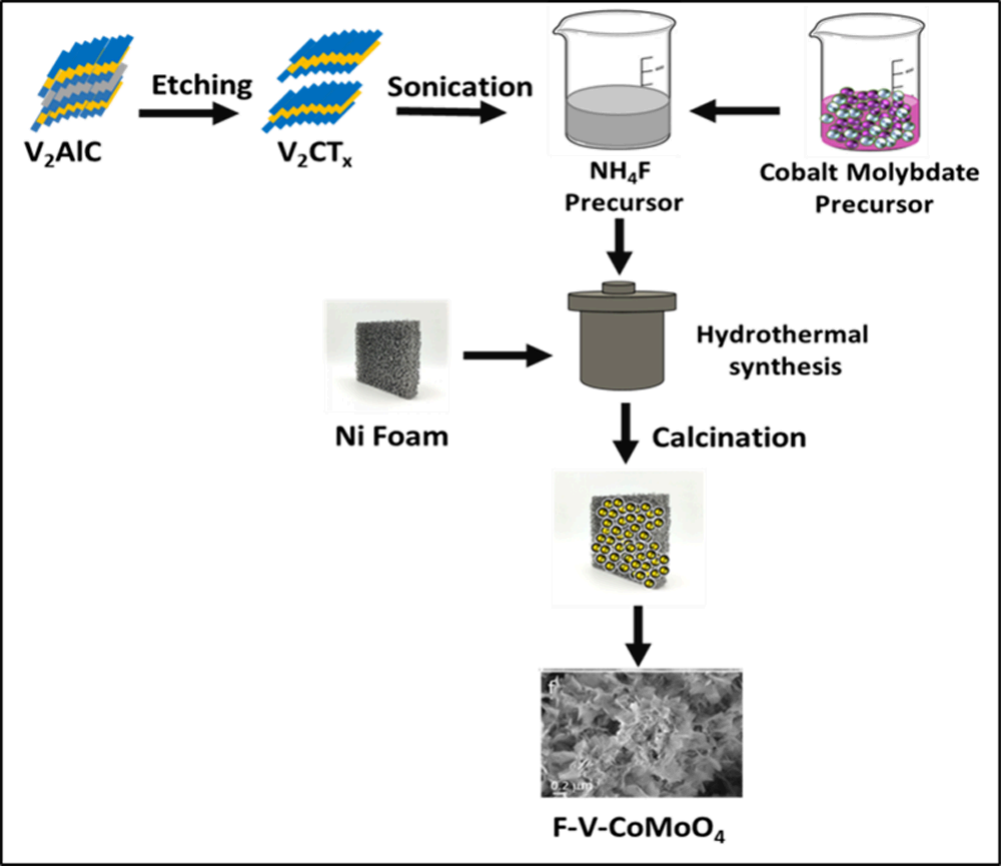
Schematic Illustration of the Synthesis Process of F-V-CoMoO_4_ on Nickel Foam

**Figure 1:**
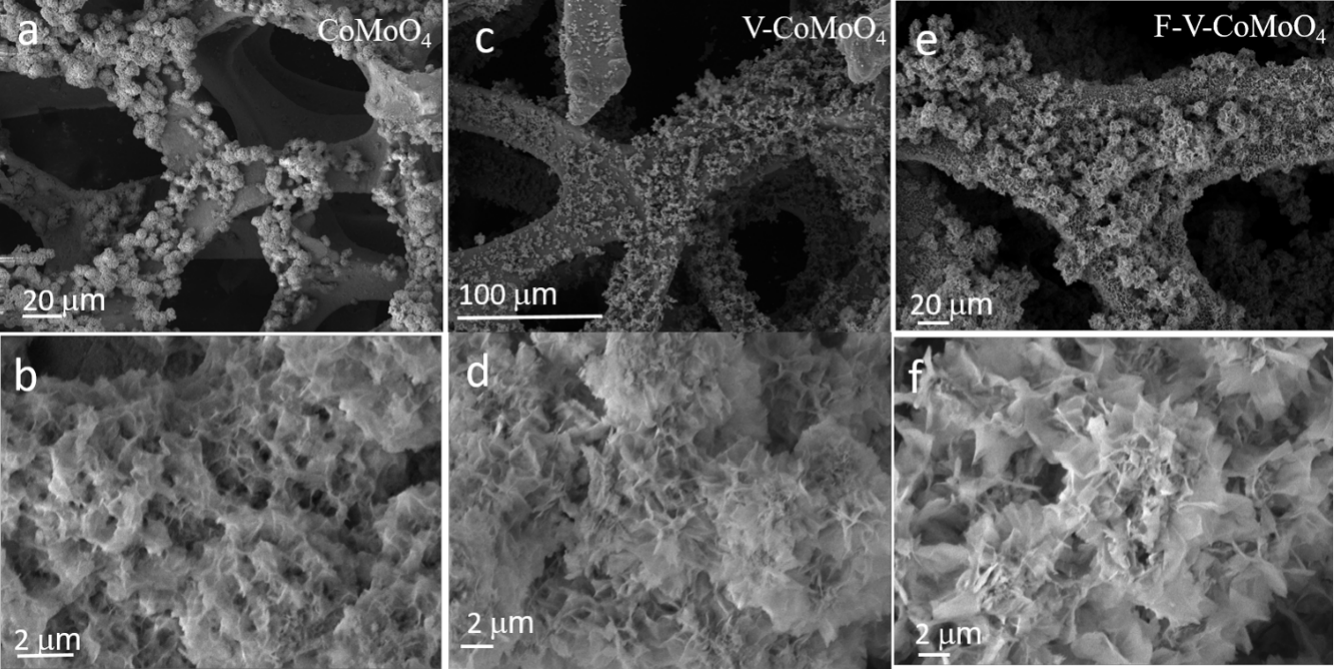
SEM images of (a,b) CoMoO_4_, (c,d) V-CoMoO_4_, and (e,f) F-V-CoMoO_4_.

The X-ray diffraction (XRD) patterns presented in Figure 2a indicate that the characteristic peaks of pristine CoMoO_4_ observed at 2θ values of 28.1, 32.8, 33.6, 38.89, 43.12, and 58.8° can be assigned to the (−311), (−222), (400), (040), (113), (−424), and (260) planes of monoclinic phase (JCPDS No. 21-0868), respectively. The phase states of V-CoMoO_4_ and F–V-@CoMoO_4_ samples are also investigated. For V-CoMoO_4_, the major diffraction peaks correspond to CoMoO_4_, with a broad peak around a 2θ value of 15°, which was attributed to the presence of carbon. No distinct peaks are observed to confirm the phases of vanadium oxide or V_2_C MXene. This indicates that the V_2_C MXene is completely oxidized, with no remaining traces of the original MXene structure or any intermediate VO_*x*_ phase detected. Additionally, we note that the oxidation of MXene can proceed through intermediate reduced vanadium species during the hydrothermal step, which is subsequently oxidized during air calcination to form VO_*x*_. However, due to their likely amorphous or low-crystallinity nature, VO_*x*_ phases may not be clearly detectable by XRD. Similarly, fluorine doping did not introduce any new phases or impurities related to the incorporation of F ions.

**Figure 2:**
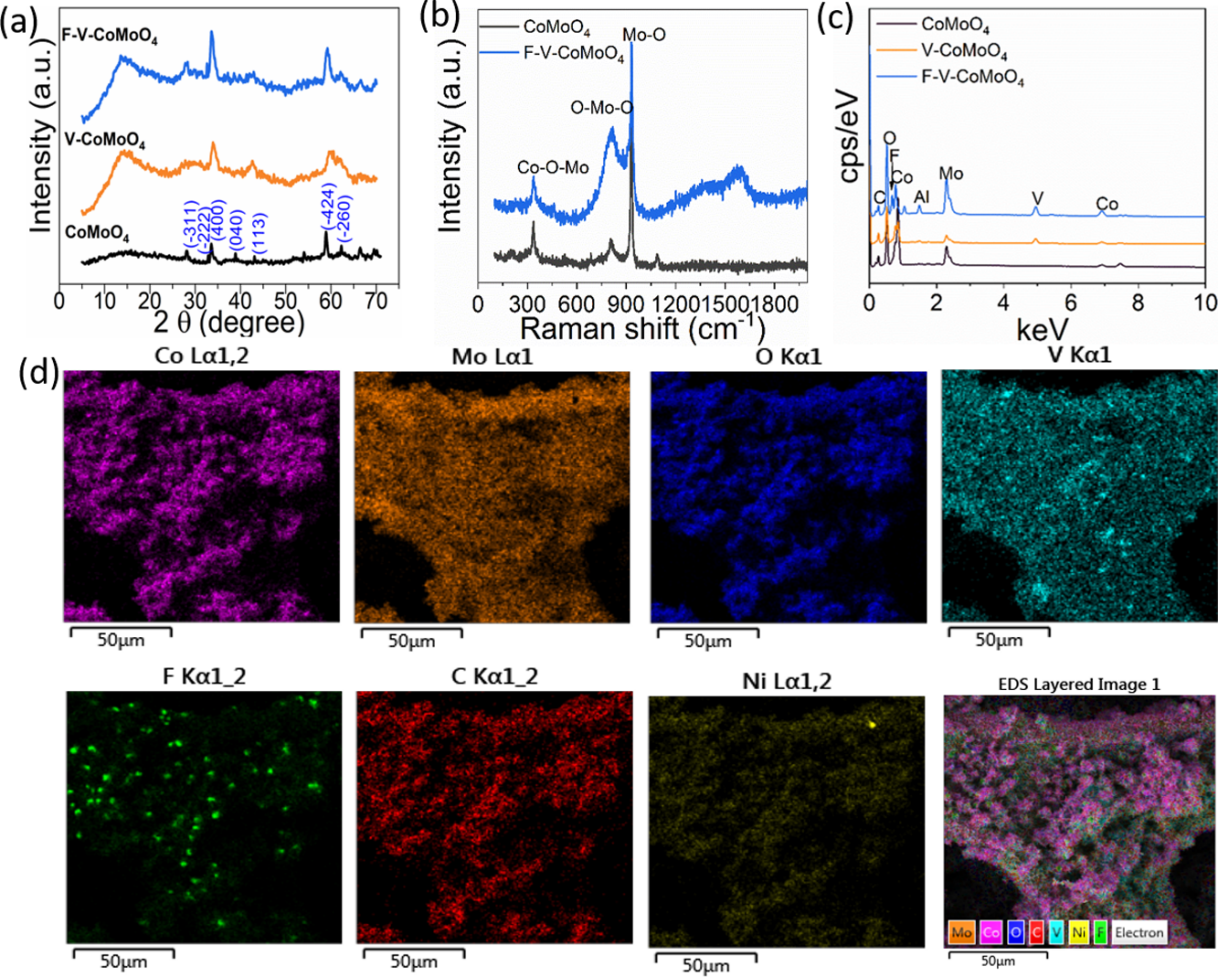
X-ray diffractograms of (a) pristine CoMoO_4_ (black), V-CoMoO_4_ (orange), and F-V-CoMoO_4_ (blue). (b) Raman spectra of CoMoO_4_, F-V-CoMoO_4_, and (c,d) EDX spectrum and elemental mapping images of F -V-CoMoO_4_, respectively.

The Raman spectra of CoMoO_4_ and F-V-CoMoO_4_ samples presented in Figure 2b exhibit the characteristic peaks at 934, 813, and 334 cm^–1^ corresponding to the symmetric/asymmetric stretching mode of the MoO_4_ tetrahedral, O–Mo–O, and Mo–O–Co bonds, respectively.^9,27^ The peak observed at approximately 1090 cm^–1^ is likely attributable to the cobalt phase, a vibrational mode that has not been previously reported in CoMoO_4_.^9^ The absence of any Raman peak shift or intensity increase upon doping with F and V atoms suggests that the band structure of CoMoO_4_ remains unchanged. This indicates that the MoO_4_ tetrahedral units and the Co–O–Mo framework are not significantly distorted after modification. Additionally, the lack of new peaks or broadening implies that no secondary phases have formed, suggesting that the dopants either weakly interact with the backbone structure or are incorporated in a way that does not alter the vibrational symmetry. This is indicative that F and V are substituting in lattice sites or occupying interstitial positions. Figure S1a,b depict the energy-dispersive X-ray spectroscopy (EDX) spectrum and the EDX elemental mapping, respectively, of the CoMoO_4_ sheets, confirming the presence of Co and Mo metals along with O, consistent with the oxide composition. The quantifiable atomic amounts of Co and Mo are nearly equal, further validating the successful formation of the CoMoO_4_ phase. Similarly, as illustrated in Figure 2c,d, the EDX analysis of the F-V-CoMoO_4_ sample reveals a uniform distribution of Co, Mo, V, and C elements. F atoms were also detected in certain areas, likely as a result of localized doping or surface adsorption.

XPS analysis was performed on the CoMoO_4_, V-CoMoO_4_, and F-V-CoMoO_4_ samples to identify their elemental compositions and the oxidation states of each constituent element. The survey spectrum, shown in Figure 3a, reveals the primary elements Co, Mo, and O, corresponding to the CoMoO_4_ phase. Additionally, the presence of doping elements V and F was also observed.

**Figure 3:**
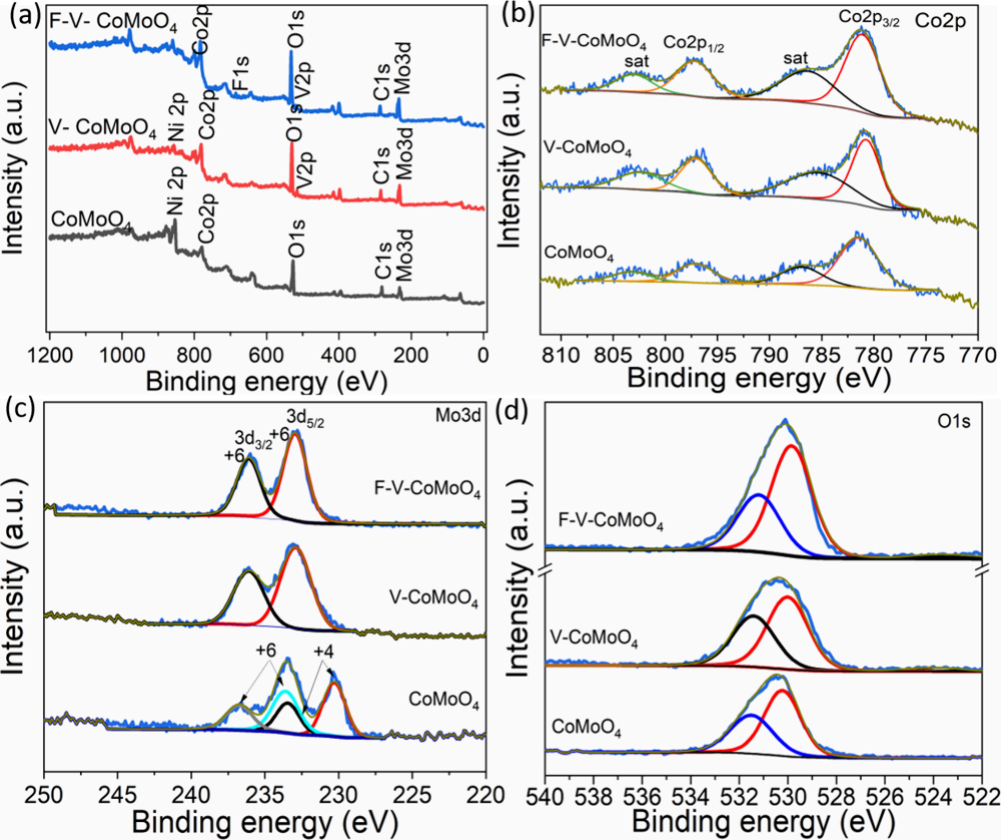
XPS spectra of CoMoO_4_, V-CoMoO_4_, and F-V-CoMoO_4_: (a) survey spectrum, (b–d) high-resolution spectra of (b) Co 2*p*, (c) Mo 3*d*, and (d) O 1*s* and V 2*p*.

The high-resolution Co *2p* spectrum of pristine CoMoO_4_ displays two distinct spin–orbit doublet peaks located at 781.4 and 797.2 eV, along with two satellite peaks at 786.9 and 803.4 eV (Figure 3b). For the V-CoMoO_4_ sample, the spin–orbit doublet peaks appear at 780.7 and 797.0 eV, with satellites observed at 785.8 and 802.5 eV. Similarly, in the F-V-CoMoO_4_ sample, the spin–orbit doublet peaks are located at 781.3 and 797.2 eV, corresponding to Co 2*p*_3/2_ and Co 2*p*_1/2_, respectively, and are accompanied by satellite peaks at 786.5 and 803.1 eV, typically attributed to cobalt oxide species.^23,24,28^ Overall, the peak shapes and positions are in good agreement with previous reports on CoMoO_4_.^29^

Furthermore, the spin–orbit splitting (Δ*E*) of ∼16 eV confirms the presence of the Co^2+^ species in the sample. Based on prior research conducted by Oku and Hirokawa,^30^ it has been established that Δ*E* values are influenced by the oxidation state, with Δ*E* about 15 eV for diamagnetic cobaltous compounds (Co^3+^) and Δ*E* of 16 eV for paramagnetic cobaltous compounds (Co^2+^). The observed Δ*E* of ∼16 eV in this study is consistent with the paramagnetic Co^2+^ configuration. Additionally, the detection of satellite peaks, which are generally absent in systems dominated by Co^3+^, exhibits additional evidence supporting the predominance of Co^2+^. These findings align the electronic state of cobalt in F-V-CoMoO_4_ and its correlation with the observed spectral features.

Figure 3c presents the high-resolution spectra of Mo 3*d*. For the CoMoO_4_ sample, the spectrum can be deconvoluted into four components located at 230.3 and 233.5 eV, corresponding to the Mo 3*d*_5/2_ and Mo 3*d*_3/2_ spin–orbit components of Mo^4+^, respectively. Additionally, a second pair of peaks located at 233.6 and 236.8 eV originates from Mo^6+^. The Δ*E* of approximately 3.2 eV aligns with the characteristic features of Mo 3d, as reported in the literature.^31,32^ Compared to the pristine sample, the F- and V-doped samples only possess the Mo^6+^ component, indicating additional oxidation of Mo during the hydrothermal synthesis. According to Table S1, the Co content increases with V doping (V-CoMoO_4_: 9.9%) compared to pristine CoMoO_4_ (6.88%), suggesting that vanadium might enhance the stabilization of Co within the structure. However, the reduction in Co content in F-V-CoMoO_4_ (8.89%), along with a similar decrease in Mo content, indicates a potential competition or redistribution of elements caused by the introduction of fluorine. This redistribution could significantly alter the electronic properties of the material. In the O 1*s* spectra (Figure 3d), two peaks at 530.22 and 531.50 eV in unmodified CoMoO_4_ are assigned to lattice oxygen and adventitious oxygen, respectively. Upon incorporation of F and V atoms in CoMoO_4_ lattices, these peaks exhibit a slight shift to lower binding energies, appearing at 529.84 and 531.84 eV in the F-V-doped sample. This shift coupled with the steady increase in oxygen content observed in Table S1, reaching its highest value in F-V-CoMoO_4_ (54.01%), suggests enhanced oxidation or the formation of more oxygen–metal bonds. In addition, the increase in oxygen amount provides more evidence that fluorine doping not only alters the surface chemistry but also favors vanadium oxidation, potentially improving the material’s oxidative capacity.^23,33^ As shown in Figure S2b, the fitted V 2*p*_3/2_ spectrum of the F-V-CoMoO_4_ sample exhibits peaks at 514.6, 515.7, and 516.76 eV, while the V 2*p*_1/2_ spectrum exhibits peaks at 522.20, 523.30, and 524.36 eV. These peaks correspond to V^3+^, V^4+^, and V^5+^ oxidation states, respectively.^34,35^ The reduced V content in F-V-CoMoO_4_ (0.57%) suggests that fluorine partially offsets the incorporation of vanadium, likely due to the interplay between these doping elements. On the surface of CoMoO_4_, different forms of VO_*x*_ species can exist—isolated, polymeric, and crystalline. Their ability to be reduced (reducibility) decreases as they become more polymerized: isolated VO_*x*_ is the easiest to reduce, followed by polymeric species, and crystalline species exhibiting the lowest reducibility.^36^ This hierarchy arises because stronger interactions between vanadium and the CoMoO_4_ support develop with higher degrees of polymerization, which progressively hinder the reduction process. Additionally, the presence of the F1*s* peak near 685.1 eV (Figure S2c) is indicative of the formation of F-metal bonds, confirming the successful insertion of F atoms into the CoMoO_4_ structure.^23,24^ Figure S3 displays the nitrogen adsorption–desorption isotherms of the synthesized samples. The BET analysis determined the specific surface areas to be 201.7, 206.8, and 140.3 m^2^/g for CoMoO_4_, V-CoMoO_4_, and F-V-CoMoO_4_, respectively. It is worth noting that a higher surface area typically enhances charge storage capacity by providing excessive electroactive sites. However, electrochemical performance is also influenced by other factors such as conductivity, pore structure, and induced defects. These can play a crucial role in improving conductivity and facilitating ion diffusion.

### 3.2 Electrochemical Performances

To evaluate the practical use of CoMoO_4_, V-CoMoO_4_, and F-V-CoMoO_4_ grown in situ on Ni foam electrodes in electrochemical energy storage, their electrochemical performance is assessed using a three-electrode system. The synthesized materials were used as the working electrode, while platinum foil and Hg/HgO served as the counter and reference electrodes, respectively. Figure 4a presents a comparative CV curve for different electrodes, carried out in a 6 M KOH aqueous electrolyte at a scan rate of 1 mV/s. The CV curves display a distinct pair of redox peaks, indicating that the capacitance primarily arises from the Faradaic redox mechanism associated with M-O bonds, where M denotes Co, Mo, V, or F. Notably, the F-V-CoMoO_4_@NF electrode exhibits the largest enclosed CV curve area, signifying a higher specific capacitance compared with other samples. This improvement can be attributed to the doping effect since F–V doping increases the area of the CV curves due to more active sites accessible for ion transport.

**Figure 4:**
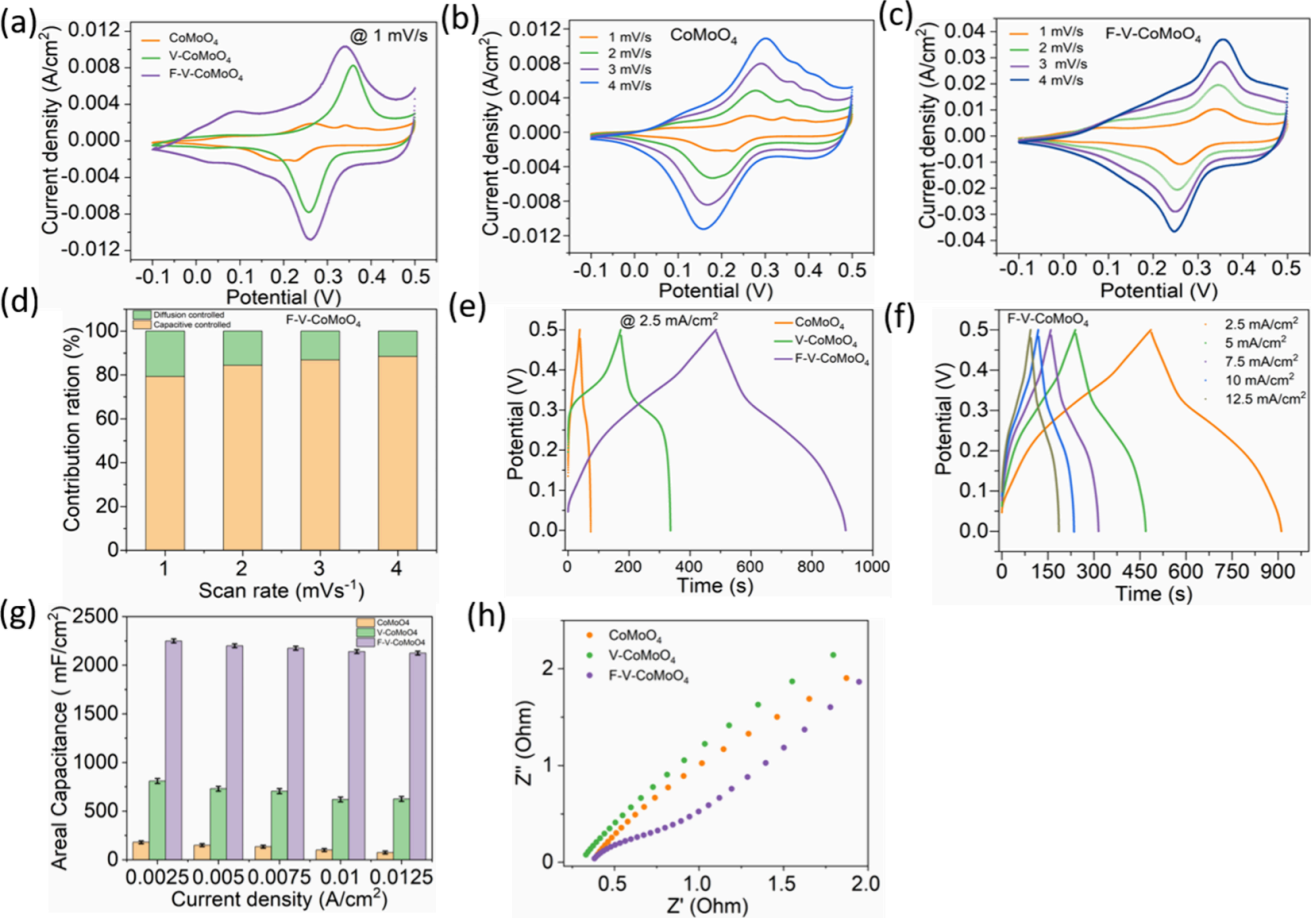
(a) CV curves of CoMoO_4_, V-CoMoO_4_, and F-V-CoMoO_4_ electrodes recorded at a scan rate of 1 mV/s. (b,c) CV curves of CoMoO_4_ and F-V-CoMoO_4_ electrodes at different scan rates ranging from 1 to 4 mV/s. (d) Proportion of capacitive- and diffusion-controlled Faradaic contribution to charge storage in F-V-CoMoO_4_ electrodes at various scan rates. (e) Comparative GCD profiles for pristine CoMoO_4_, V-CoMoO_4_, and F-V-CoMoO_4_ electrodes. (f) GCD profiles of the F-V-CoMoO_4_ electrode at different current densities. (g) Corresponding areal capacitances at various current densities. (h) Comparison of Nyquist diagrams of CoMoO_4_, V-CoMoO_4_, and F-V-CoMoO_4_.

Figures 4b and S4a show the CV curves of the CoMoO_4_ electrode recorded at scan rates ranging from 1 to 30 mV/s. In all curves, a distinct pair of redox peaks are observed, corresponding to the Co^2+^/Co^3+^ and Co^3+^/Co^4+^ redox, along with the oxidation of Mo ions. Notably, while Mo ions typically do not participate in the reduction process under normal conditions, the introduction of structural defects can create active sites that enable the reduction of Mo^6+^ to Mo^5+7, 37^. Furthermore, as the scan rate increases, the current values of both oxidation and reduction peaks are enhanced and shifted, indicating the occurrence of quasi-reversible redox reactions at the electrode–electrolyte interface. The shift in the peak position is attributed to charge diffusion polarization within the electrode. Similarly, the CV curves of V-CoMoO_4_ (Figure S4b) and F-V-CoMoO_4_ (Figure 4c) electrodes at different scan rates show a linear increase in current density, along with a noticeable shift in the redox peak positions. This behavior indicates improved charge transfer kinetics and enhanced electrochemical activity due to the incorporation of V and F dopants, which likely introduce additional active sites and facilitate ion diffusion.

The total capacitance of the electrode consists of two distinct contributions: surface-controlled capacitive behavior and diffusion-controlled charge storage. The proportion of diffusion-controlled capacitance can be quantitatively evaluated using Dunn’s equation:

$$i=k_{1}\nu +k_{2}\nu^{1/2}$$ 5

where *i* belongs to the current (A), ν denotes the scan rate of the CV test (mV/s), *k*_1_ represents the surface-controlled capacitive contribution, and *k*_2_ corresponds to the diffusion-controlled contribution. To obtain these parameters, a linear plot $i/\nu^{1/2}$ versus $\nu^{1/2}$ is adopted (Figure S4c,d), where the slope and intercept provide the *k*_1_ and *k*_2_, respectively.^38^ As illustrated in Figure 4d, the F-V-CoMoO_4_ electrode demonstrates the highest surface-controlled capacitive contribution, with values of 80, 84, 86, and 88% at scan rates of 1, 2, 3, and 4 mV/s, respectively. This finding suggests that the capacitive behavior dominates, while the diffusion contribution gradually diminished inversely with increasing scan rate due to the limited time available for the ion diffusion reaction. The capacitive contribution can also be quantitatively analyzed using the equation *i* = *a*ν^b^, where *i* represents the current response, and ν denotes the scan rate. Here, *a* and *b* are adjustable constants, with *b* obtained from the slope of log *i* versus log ν.^39^ In the case of the F-V-CoMoO_4_ electrode (Figure S4f,g), the calculated b values are 0.92 for the oxidation peak and 0.87 for the reduction peak, confirming a surface-controlled reaction mechanism.

Figure 4e presents comparative GCD plots obtained at a current density of 2.5 mA/cm^2^ (1 A/g) to evaluate the capacitance characteristics of the electrodes within the potential range of 0–0.5 V. Notably, the presence of a voltage plateau in the GCD curve indicates the occurrence of oxidation–reduction reactions during the electrochemical process, consistent with the CV characterization results. Moreover, the F-V-CoMoO_4_ electrode exhibits a significantly longer discharge time compared to both pristine and V-doped CoMoO_4_ electrodes.

To evaluate the capacitive properties of F-V-CoMoO_4_, GCD measurements are conducted with the fabricated electrodes and measured at different current densities ranging from 2.5 to 12.5 mA/cm^2^ (Figure 4f). The symmetrical shape of the GCD curves indicates the reversibility of the faradic redox reaction, with a high Coulombic efficiency of 93%. We further analyzed the Coulombic efficiency over a range of current densities, which shows a decrease for pristine CoMoO_4_, as presented in Figure S4h. However, the Coulombic efficiency remains stable at around 93% for the F-V-CoMoO_4_ electrode. The areal capacitance results are illustrated in Figure 4g, where the CoMoO_4_ and V-doped samples exhibited capacitance values of 180 and 810 mF/cm^2^, respectively, at a current density of 2.5 mA/cm^2^. In contrast, the F-V-CoMoO_4_ electrode demonstrated significantly higher capacitance, ranging from 2125 mF/cm^2^ at 12.5 mA/cm^2^ (Csp = 850 F/g at 5 A/g) to 2250 mF/cm^2^ at 2.5 mA/cm^2^ (Csp = 900 F/g at 1 A/g), which achieved the highest capacitance among the evaluated material. The capacitive properties of F-V-CoMoO_4_ are better than those reported for some conventional modified CoMoO_4_-based electrodes, including CoMoO_4_@reduced graphene composites, as shown in Table S2. This substantial capacitance performance underscores the superior electrochemical properties of the F-V-CoMoO_4_ electrode and can be attributed to the intricately developed morphology, the abundance of exposed redox-active sites, the synergistic effect of a coexistence of metallic ion redox pair, and the potential of F–V in modulating the redox reaction.

Electrochemical impedance spectroscopy (EIS) is essential for evaluating the intrinsic resistance at the electrode–electrolyte interface. By applying a sinusoidal perturbation at zero bias, the impedance is measured as a function of frequency in the range of 10 mHz to 100 kHz. Figure 4h presents the Nyquist plots, where the F-V-CoMoO_4_ electrode shows a noticeably reduced semicircle radius in the high-frequency region. This reduction indicates effective charge transfer at the semiconductor/Ni foam interface. Additionally, the emergence of this semicircle underscores the critical impact of interface effects on the electrical properties of the involved electrodes.

### 3.3 Evaluation of F-V-CoMoO_4_//AC Asymmetric Supercapacitors in PVA/KOH Gel Electrolytes

An asymmetric supercapacitor (ASC) device is fabricated using a binder-free F-V-CoMoO_4_ electrode directly grown on Ni foam to further evaluate its electrochemical properties and suitability for practical application. The device is assembled with activated carbon as the negative electrode, while the microfibers serve as separators. Additionally, an all-solid-state asymmetric device (F-V-CoMoO_4_@NF//AC@NF) is constructed using a PVA-KOH gel electrolyte to assess its performance at room temperature, as illustrated in Figure 5a.

**Figure 5:**
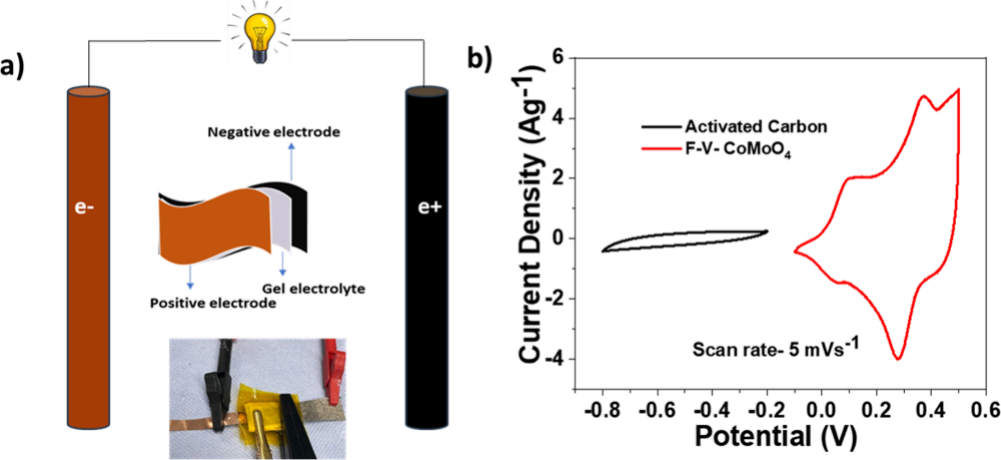
(a) Schematic presentation of ASC device fabrication. (b) Cyclic voltammetry curves of activated carbon and the F-V-CoMoO_4_ material at a scan rate of 5 mVs^–1^.

Prior to the analysis, the electrochemical properties of the activated carbon were evaluated using the 6 M KOH electrolyte, as shown in Figure 5b. The potential window of the F-V-CoMoO_4_ electrode ranges from −0.1 to 0.5 V, while that of the AC/CC electrode extends from −0.8 to 0.2 V. This broad potential range indicates that the voltage of the F-V-CoMoO_4_ /CC//AC/CC asymmetric device can be extended to a higher potential, therefore ameliorating its energy storage capacity.

Furthermore, the mass loading of the positive electrode (F-V-CoMoO_4_) is balanced through the following charge balance equation:

$$\frac{m^{+}}{m^{-}}=\frac{c^{-}V^{-}}{c^{+}V^{+}}$$ 6

where, *m* ^+^ and *m* ^–^ correspond to the mass loading on positive and negative electrodes, while the *c* ^+^ and *c* ^–^ are the specific capacitances of F-V-CoMoO_4_ and activated carbon in F g^–1^, respectively. *V* ^+^ presents the applied positive potential for F-V-CoMoO_4_, and *V* ^–^ is the supplied potential of activated carbon.^37^

Figure 6a presents the CV curves recorded at different applied voltages ranging from 0 to 1.4 V–2 V, all measured at a constant sweep rate of 20 mV s^–1^. The obtained CV curves remain consistent across various potential windows, which reflect a stable electrochemical behavior. The ASC device exhibits nearly symmetric curves up to 1.5 V, suggesting good capacitive performance. However, oxygen evolution reactions (OERs) occur beyond this voltage threshold, defining 0–1.5 V as the optimal working potential for the ASC device. In addition, CV measurements conducted at a scan rate ranging from 1 to 50 mVs^–1^ (Figure 6b), demonstrating that the curves retain their shape, confirming the stability of the device and its ability to maintain performance without deteriorating rate capability. At a lower scan rate of 1–3 mVs^–1^, distinct redox peaks are observed in the CV curves, indicative of the faradaic process associated with a slower kinetic reaction. However, as the scan rate increases from 10 to 50 mVs^–1^, the redox peaks gradually diminish and become invisible due to the faster kinetic reaction. At these high scan rates, the CV curves exhibit quasi-reversible behavior. The symmetric GCD curves are obtained up to a potential of 1.5 V, showing good Coulombic efficiency. Beyond this potential, efficiency decreases with increasing voltage, likely due to side reactions or electrolyte decomposition. Additionally, different GCD curves are obtained at various current densities, also demonstrating good Coulombic efficiency, as given in the inset of Figure 6c. The device demonstrates excellent reversible cycling stability, maintaining 100% capacitance retention up to 2000 cycles at a current density of 1 A g^–1^. Beyond this, for up to 3000 cycles, approximately 94% of the initial capacitance is retained, as shown in Figure 6d. The device achieves a maximum specific capacitance of 36.2 F g^–1^ at a current density of 0.3 A g^–1^, showing excellent Coulombic efficiency. This high symmetry in the charge–discharge curves further confirms the superior electrochemical performance and stability of the F-V-CoMoO_4_@NF//AC@NF device. The specific capacitances of the F-V-CoMoO_4_@NF//AC@NF device at different current densities are calculated using eq 2 and presented in Figure 6d (inset), along with the Coulombic efficiency at different current densities. At a current density below 0.3 A g^–1^, the charge–discharge curve loses its symmetry, and the Coulombic efficiency decreases. Notably, all GCD curves from 2 to 0.3 A g^–1^ exhibit battery-type behavior. A maximum power density of 1500 W kg^–1^ is observed, while the ASC device delivers a high specific energy density of 11.5 W kg^–1^ and a specific power density of 225 Whkg^–1^ at a current density of 0.3 A g^–1^. These values are illustrated and compared to other modified CoMoO_4_-based materials in the Ragone plot presented in Figure 6e.^14,44–46^ The electrochemical (EIS) behaviors of the ASC device have been studied and are shown in Figure 6f. The obtained Nyquist plots are fitted, and the corresponding equivalent circuit diagram is given in the inset of Figure 6f. The high-frequency region provides a small internal resistance of 0.58 Ω and an equivalent series resistance of 1.66 Ω, indicating low internal resistance and efficient charge transport. Moreover, the Nyquist plot is nearly parallel to the *Y*-axis, suggesting a diffusion-limited electron transfer process. A small Warburg resistance is also observed, along with the capacitance behavior. Furthermore, the EIS analysis confirms the enhanced electrochemical stability of the F-V-CoMoO_4_@NF//AC@NF device.^37,40^ The excellent electrochemical performance of the ASC device can be ascribed to several factors: (i) the wide applied potential window of 1.7 V significantly increases the energy density of supercapacitors; (ii) CoMoO_4_ attributes to the pseudo capacitance behavior, while the F and V may support the EDLC capacitance with high conductivity; (iii) the strong interaction between F–V with CoMoO_4_ ensures the remarkable cycling and rate performance of the F-V-CoMoO_4_@NF//AC@NF device; and (iv) doping of F and V meaningfully improves the electrochemical performance owing to the porous structure that facilitates ion transport and supports a rapid redox reaction.^41,42^ Nevertheless, the presence of F and V contributes to the high supercapacitor performance of the CoMoO_4_ material. Morphological evaluation of the electrodes after extensive cycling can yield valuable structural and electrochemical insights. Following long-term cycling (3000 cycles), the F-V-CoMoO_4_ nanosheets retained their structural architectures with minimal deformation, as confirmed by SEM imaging in Figure S5.

**Figure 6:**
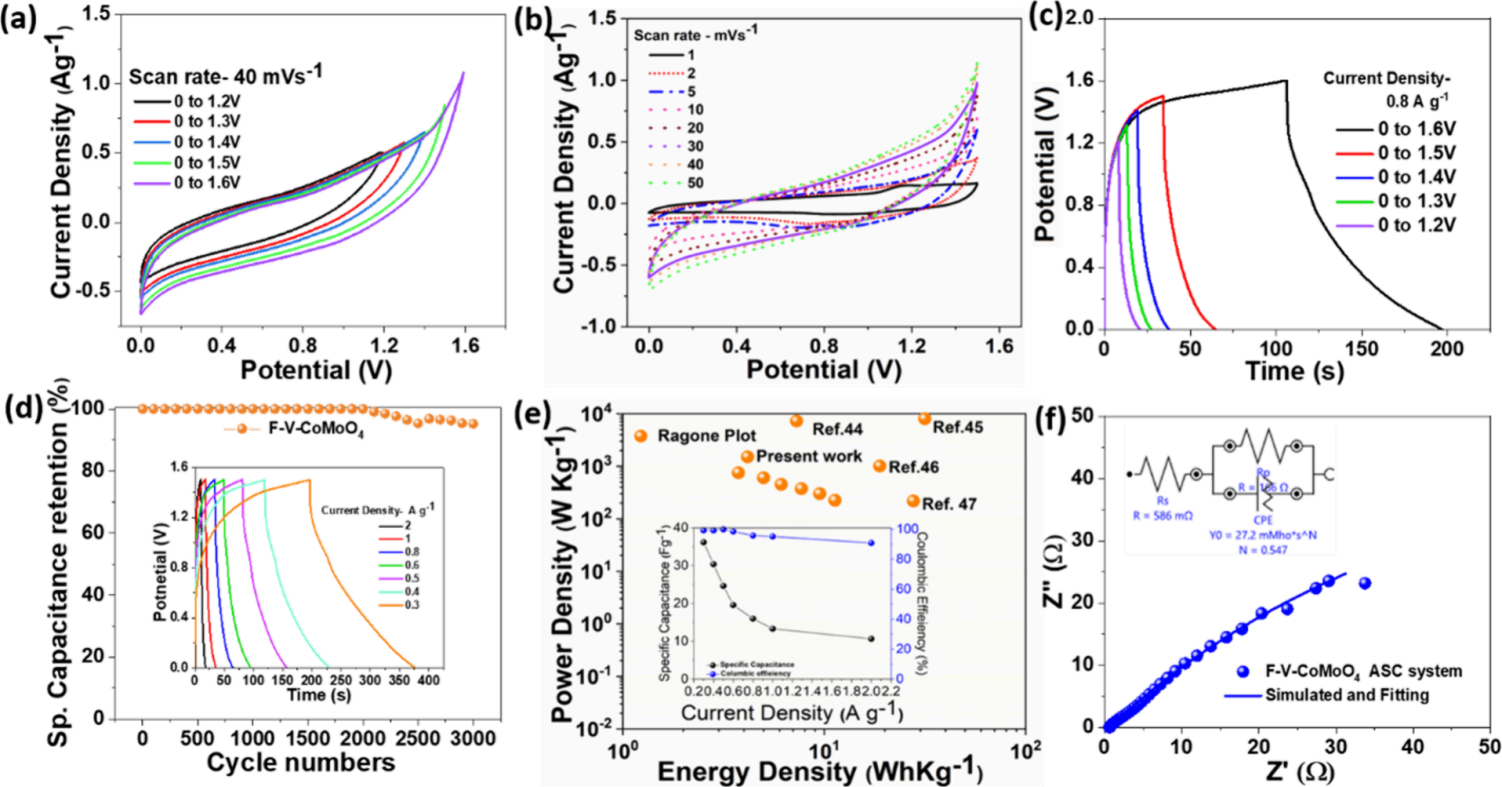
Asymmetric system (F-V-CoMoO_4_@NF//AC@NF): (a) CV curves at different potential windows and at a scan rate of 40 mV s^–1^, (b) CV curves at different scan rates from 1 to 50 mV s^–1^, (c) GCD curves at a current density of 0.8 A g^–1^, (d) cycle stability up to 3000 cycles and GCD curves at current densities ranging from 2 to 0.8 A g^–1^ (inset), (e) Ragone plot, specific capacitance values in F g^–1^ and Coulombic efficiencies in % at different current densities (inset), and (f) Nyquist plot with the circuit diagram (inset).

The optimized structures of pristine and V- and F-doped CoMoO_4_ are shown in Figure 7a,b. Doping leads to slight lattice distortions, indicating structural stability upon substitution. Both structures showed a zero band gap, indicating that their metal characteristics were suitable for fast electron transport in catalysis.^43^ The projected density of states (PDOS) analysis reveals that V and F doping significantly alters the electronic structure by introducing impurity states near the Fermi level, enhancing charge transfer. The V incorporation from V_2_C leads to hybridization with Mo orbitals, contributing to enhanced electronic conductivity (Figure 7c,d). The work function, a crucial parameter for electron emission and surface reactivity, was computed for both pristine and doped CoMoO_4_. The pristine CoMoO_4_ exhibits a work function of 6.3 eV, whereas the V, F-doped CoMoO_4_ shows a reduced work function of 5.6 eV. This decrease indicates an enhanced electron transfer capability, which can facilitate better electrochemical performance and is consistent with the obtained enhancement of the experimental measurements.

**Figure 7:**
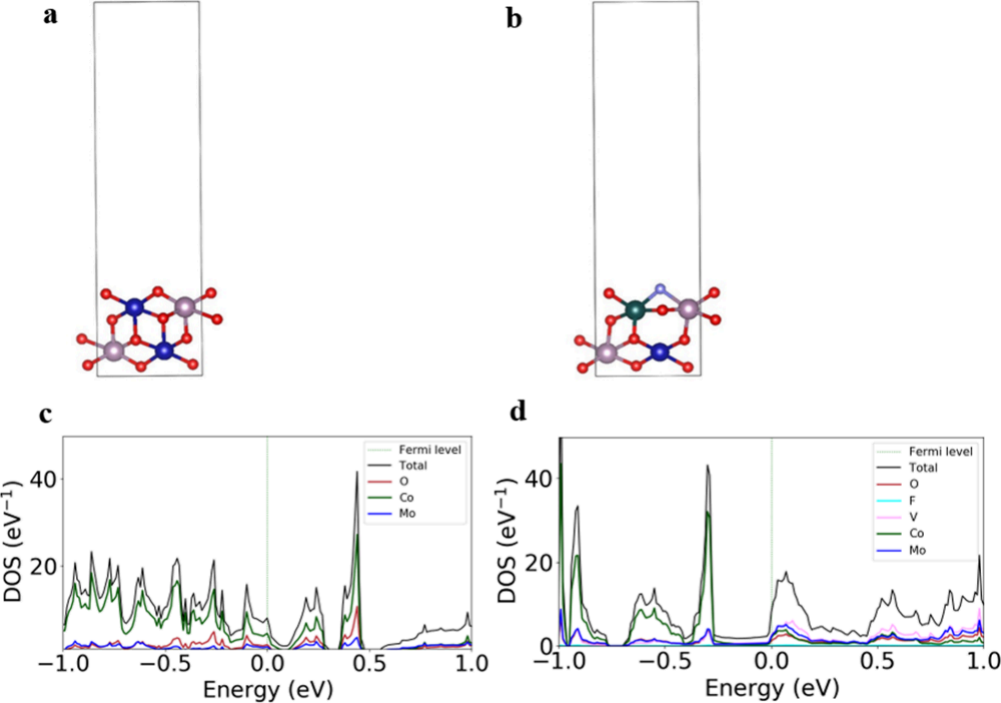
Optimized structures of (a) CoMoO_4_ and (b) V, F-doped CoMoO_4_. Projected density of states of (c) CoMoO_4_ and (d) V, F-doped CoMoO_4_.

The increased DOS near the Fermi level and lowered work function suggest improved conductivity and ion transport, making doped CoMoO_4_ an excellent candidate for supercapacitor electrodes. These insights provide a theoretical foundation for experimental validation.

## 4 Conclusions

In summary, CoMoO_4_, V-CoMoO_4_, and F-V-CoMoO_4_ samples are successfully synthesized via a hydrothermal method, followed by thermal calcination at 350 °C. Overall, the combined effect of F and V doping alters the structural properties of CoMoO_4_ and induces defects such as oxygen vacancies and interstitials that significantly enhance its electrochemical performance. Especially, the F-V-CoMoO_4_ electrode achieves an areal capacitance of approximately 2250 mF/cm^2^ at a current density of 2.5 mA/cm^2^ (Csp = 900 F/g at 5 A/g), outperforming the pristine CoMoO_4_ (180 mF/cm^2^, 72 F/g) and V-doped CoMoO_4_ (810 mF/cm^2^, 324 F/g). Additionally, an asymmetric supercapacitor is assembled using an F-V-CoMoO_4_@NF//AC@NF device, demonstrating the redox behavior of the material with excellent cycling stability, and 100% capacity retention after 2000 cycles is achieved at a current density of 1A g^–1^. Furthermore, the device exhibits a high specific energy density of 11.5 Whkg^–1^ and a specific power density of 225 Wkg^–1^ at a current density of 0.3 A g^–1^. V and F doping in CoMoO_4_, as revealed by DFT calculations, significantly enhances its electronic properties by introducing impurity states near the Fermi level and reducing the work function, leading to improved conductivity and charge transfer. This result paves the way for further exploration and development of modified CoMoO_4_-based electrodes for efficient electrochemical energy storage systems.

## Supplementary Material



## Data Availability

The data sets generated and/or analyzed during the current study are available in the Zenodo repository: https://zenodo.org/records/15303730.

## Notes

Experimental details, characterization methods including SEM images; EDX spectrum; elemental mapping images; high-resolution spectra of C 1s, V 2p, and F 1s; nitrogen adsorption–desorption isotherm spectra of CoMoO_4_, V-CoMoO_4_, and F-V-CoMoO4; CV curves of CoMoO_4_ and V-CoMoO_4_; plots of $i/\nu^{1/2}$ versus $\nu^{1/2}$ adopted to calculate *k*1 and *k*2 at various potentials for the F-V-CoMoO_4_ electrode; proportion of capacitive and diffusion-controlled Faradaic contribution to charge storage in the CoMoO_4_ electrode at various scan rates; log­(*i*) versus log­(ν) plots for the F-V-CoMoO_4_ electrode using oxidation and reduction peaks, Coulombic efficiency rate of pristine and F–V-doped CoMoO_4_ at different applied current densities; SEM image of the F-V-CoMoO_4_ electrode after 3000 cycles of GCD**;** atomic percentages (at.%) of surface elements in pure CoMoO_4_, V- CoMoO_4_, and F–V- CoMoO_4_ samples as obtained from the XPS analysis and comparison of electrochemical performance enhancement of the synthesized F-V-CoMoO_4_-based electrode with previously reported modified CoMoO_4_ in a three electrodes system (Table S2) (PDF)
