# Supplementary material for: Enhanced Electrochemical Performance of Binder-Free Fluorine–Vanadium-Doped CoMoO4 Nanosheets via In Situ MXene Integration for Energy Storage Applications
Source: ACS Appl Energy Mater. 2025 Jul 22;8(15):11513–23. doi: 10.1021/acsaem.5c01660 (PMC12345410; doi:10.1021/acsaem.5c01660)
Supplement: Supplementary file 1 [file ae5c01660_si_001.pdf]

Supporting Information

Enhanced Electrochemical Performance of Binder-Free Fluorine- Vanadium-Doped $CoMoO_4$ Nanosheets via In Situ MXene Integration for Energy Storage Applications

Monaam Benali*, Rasmita Barik, Rui Gusmão, Jan Luxa, Piotr W. Zabierowski, Amutha Subramani, Bing Wu, Zdeněk Sofer*

[1] Department of Inorganic Chemistry, University of Chemistry and Technology Prague, Technicka 5, 166 28 Prague 6, Czech Republic

*To whom correspondence should be addressed: Dr. Monaam Benali, email: benalim@vscht.cz
Prof. Zdenek Sofer, email: zdenek.sofer@vscht.cz

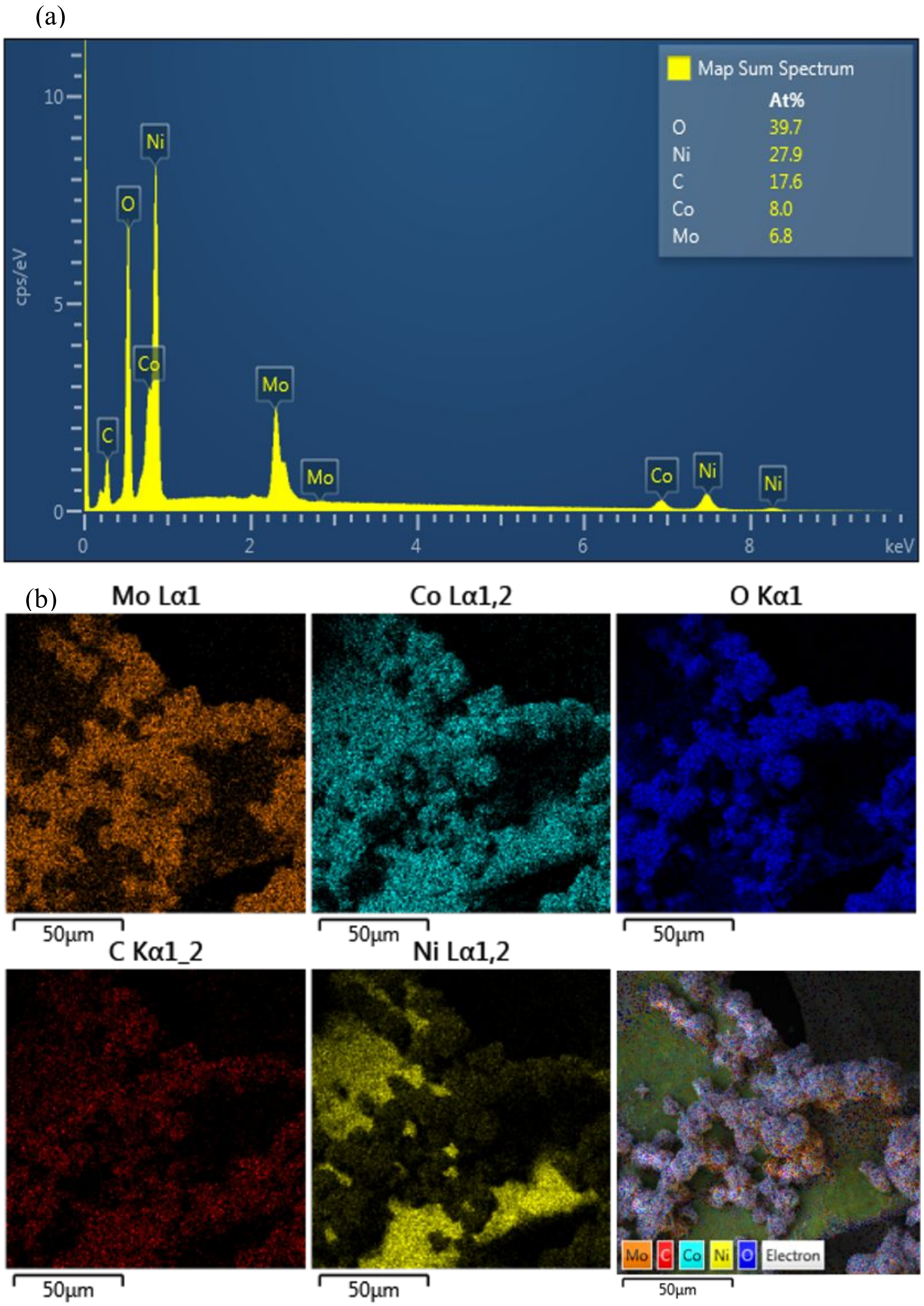

Figure S1. Energy dispersive spectrum **(a)** and elemental mapping images **(b)** of $CoMoO_4$.

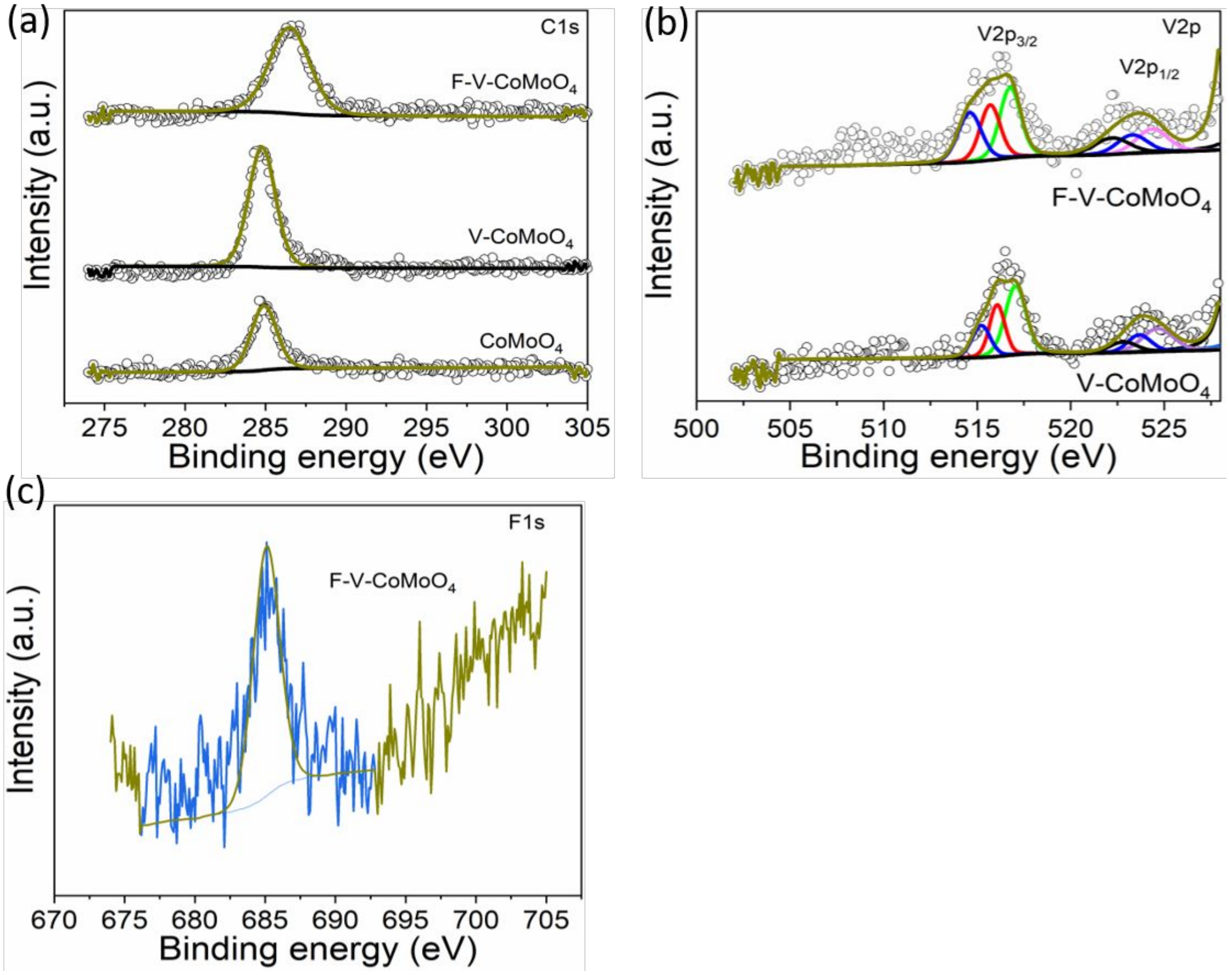

Figure S2. XPS spectra of $CoMoO_4$, V-$CoMoO_4$ and F-V-$CoMoO_4$: a) high-resolution spectra of C 1s, V 2p, and F 1s, respectively.

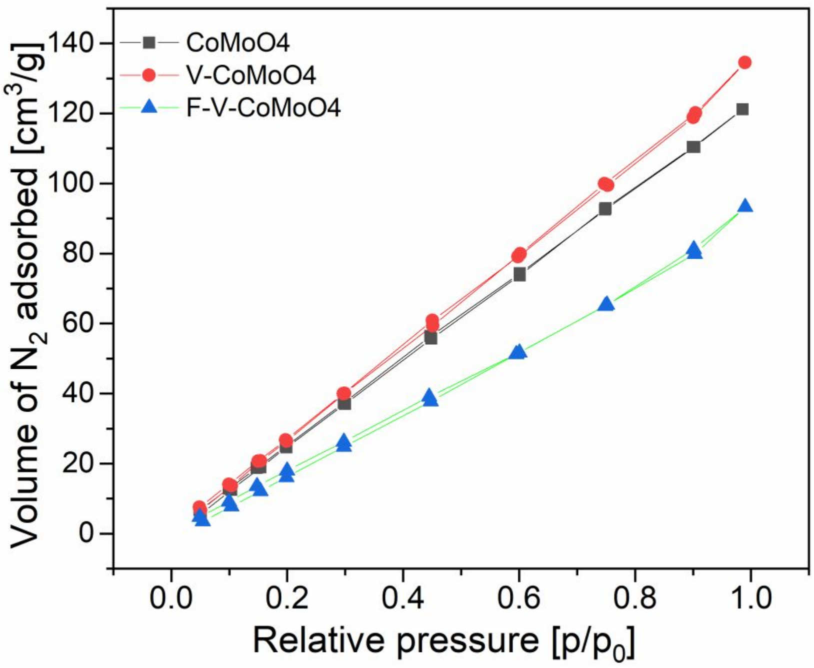

Figure S3. Nitrogen adsorption-desorption isotherm spectra of $CoMoO_4$, V-$CoMoO_4$ and F-V-$CoMoO_4$

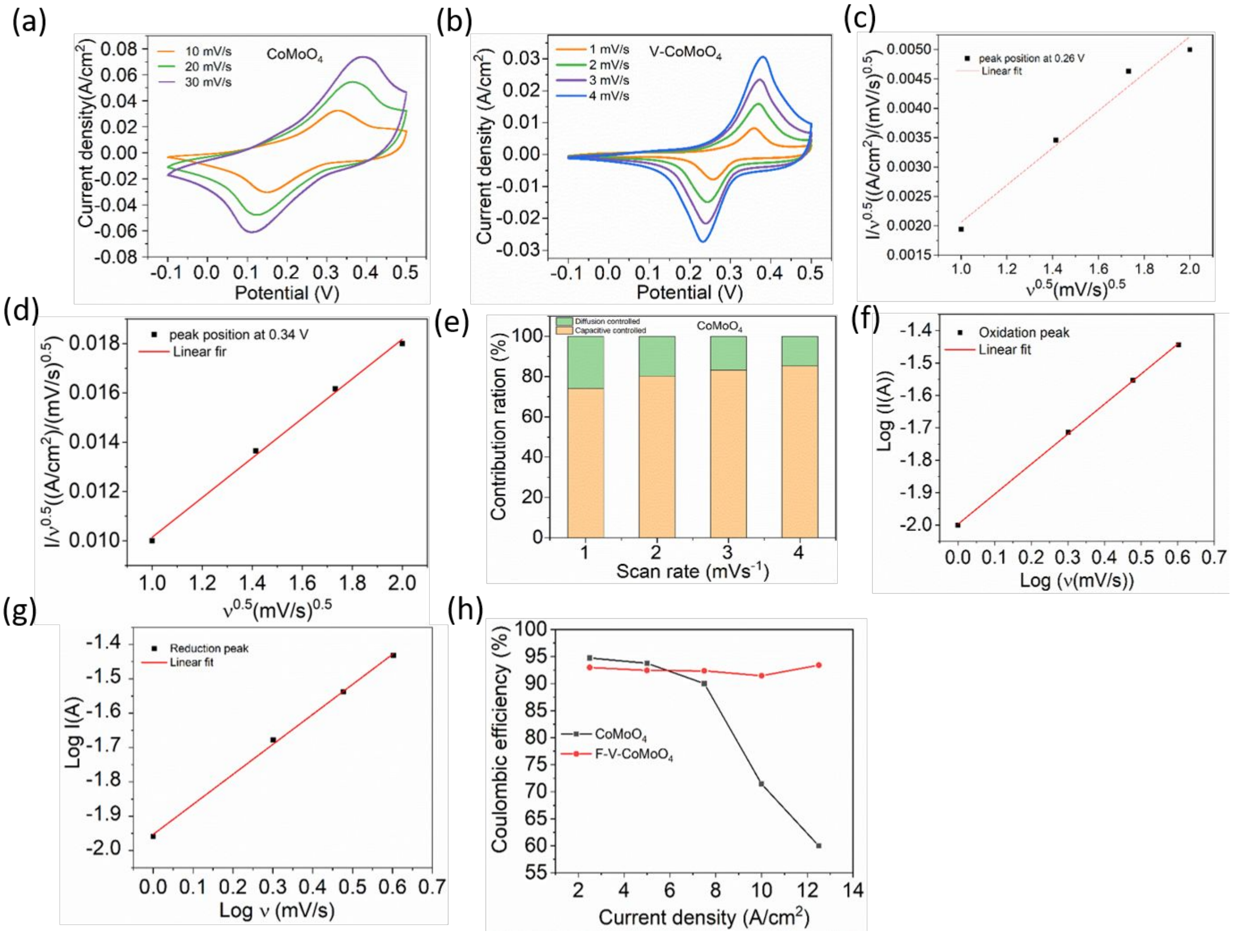

Figure S4. **(a)** and **(b)** CV curves of $CoMoO_4$, V-$CoMoO_4$, respectively, at a scan rate of 1 mV/s. **(c)** and **(d)** Plots of i/$\nu^{\frac{1}{2}}$ versus $\nu^{\frac{1}{2}}$ were adopted to calculate k1 and k2 at various potential for the F-V-$CoMoO_4$ electrode. **(e)** Proportion of capacitive and diffusion-controlled Faradaic contribution to charge storage in $CoMoO_4$ electrode at various scan rate. **(f)** and **(g)** Log(i) versus Log(ν) plots for the F-V-$CoMoO_4$ electrode using oxidation and reduction peak. **(h)** Coulombic efficiency rate of pristine and F-V-doped CoMoO4 at different applied current density.

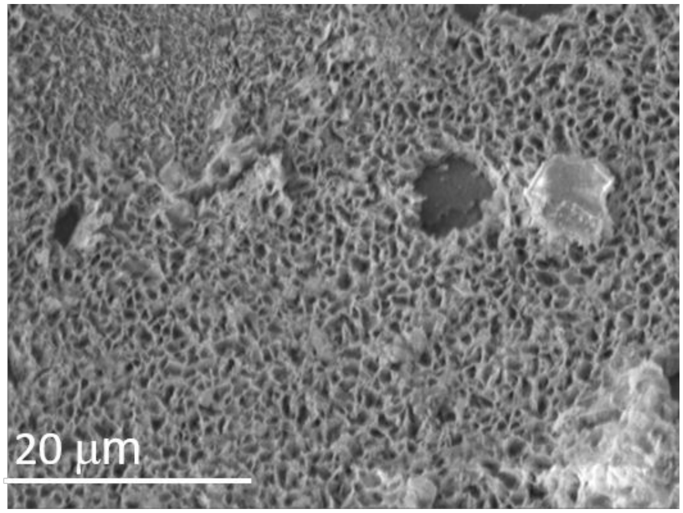

Figure S5. SEM image of F-V-$CoMoO_4$ electrode after 3000 cyles of GCD.

Table S1. Atomic percentages (at.%) of surface elements in pure $CoMoO_4$, V- $CoMoO_4$, and F-V- $CoMoO_4$ samples as obtained from the XPS analysis.

	$CoMoO_4$	V- $CoMoO_4$	F-V- $CoMoO_4$
Co2p	6.88	9.90	8.89
Mo3d	5.68	8.12	8.48
O1s	47.63	48.79	54.01
C1s	30.44	30.68	24.15
V2p		0.96	0.57
F			1.47

Table S2. Comparison of electrochemical performance enhancement of the synthesized F-V-$CoMoO_4$ –based electrode with previously reported modified $CoMoO_4$ in a three electrodes system.

Cathode based Materials	Current density	Specific capacitance	Ref.
$CoMoO_4$@CC	1 A/g	644 F/g	[1]
$CoMoO_4$@Ni Foam	1 A/g	549 F/g	[2]
Worm-like $CoMoO_4$@expanded graphene	1 A/g	1359 F/g	[2]
$CoMoO_4$@ reduced graphene oxide	1 A/g	856.2 F/g	[3]
$CoMoO_4$.0.9 H_2O nanorods@ reduced graphene oxide	1 A/g	802 F/g	[4]
$CoMoO_4$-$Ti_3C_2T_x$@NF	1 A/g	870 F/g	[5]
F-V-$CoMoO_4$	1 A/g (mass loading 2.5 mg/cm^2 ± 0.2 mg/cm^2)	900 F/g	This work

References

1. Chen, C.; Deng, H.; Wang, C.; Luo, W.; Huang, D.; Jin, T., Petal-like $CoMoO_4$ Clusters Grown on Carbon Cloth as a Binder-Free Electrode for Supercapacitor Application. *ACS Omega* **2021,** *6* (30), 19616-19622.
2. Li, F.; Huang, Z.; Hu, R.; Chen, W.; Liu, Y.; Qi, X., Self-assembled $CoMoO_4$/3D worm-like expanded graphite composites for high performance supercapacitors. *J.Power Sources* **2025,** *632*, 236305.
3. Jinlong, L.; Meng, Y.; Suzuki, K.; Miura, H., Synthesis of $CoMoO_4$@ RGO nanocomposites as high-performance supercapacitor electrodes. *Microporous Mesoporous Mater.* **2017,** *242*, 264-270.
4. Xu, K.; Chao, J.; Li, W.; Liu, Q.; Wang, Z.; Liu, X.; Zou, R.; Hu, J., $CoMoO_4$· 0.9 H_2O nanorods grown on reduced graphene oxide as advanced electrochemical pseudocapacitor materials. *RSC Adv.* **2014,** *4* (65), 34307-34314.
5. Wei, X.; Cai, M.; Yuan, F.; Li, C.; Huang, H.; Xu, S.; Liang, X.; Zhou, W.; Guo, J., Construction of $CoMoO_4$ nanosheets arrays modified by $Ti_3C_2T_x$ MXene and their enhanced charge storage performance for hybrid supercapacitor. *Colloids Surf. A Physicochem. Eng. Asp.* **2023,** *658*, 130637.
